# Roles of Extracellular Polysaccharides and Biofilm Formation in Heavy Metal Resistance of Rhizobia

**DOI:** 10.3390/ma9060418

**Published:** 2016-05-26

**Authors:** Natalia Nocelli, Pablo C. Bogino, Erika Banchio, Walter Giordano

**Affiliations:** Departamento de Biología Molecular, Facultad de Ciencias Exactas, Físico-Químicas y Naturales, Universidad Nacional de Río Cuarto, Río Cuarto, Córdoba X5804BYA, Argentina; nnocelli@exa.unrc.edu.ar (N.N.); pbogino@exa.unrc.edu.ar (P.C.B.); ebanchio@exa.unrc.edu.ar (E.B.)

**Keywords:** *S. meliloti*, exopolysaccharides, biofilm, co-culture, bacterial rescue, metal toxicity

## Abstract

Bacterial surface components and extracellular compounds, particularly flagella, lipopolysaccharides (LPSs), and exopolysaccharides (EPSs), in combination with environmental signals and quorum-sensing signals, play crucial roles in bacterial autoaggregation, biofilm development, survival, and host colonization. The nitrogen-fixing species *Sinorhizobium meliloti* (*S. meliloti*) produces two symbiosis-promoting EPSs: succinoglycan (or EPS I) and galactoglucan (or EPS II). Studies of the *S.* *meliloti/*alfalfa symbiosis model system have revealed numerous biological functions of EPSs, including host specificity, participation in early stages of host plant infection, signaling molecule during plant development, and (most importantly) protection from environmental stresses. We evaluated functions of EPSs in bacterial resistance to heavy metals and metalloids, which are known to affect various biological processes. Heavy metal resistance, biofilm production, and co-culture were tested in the context of previous studies by our group. A range of mercury (Hg II) and arsenic (As III) concentrations were applied to *S. meliloti* wild type strain and to mutant strains defective in EPS I and EPS II. The EPS production mutants were generally most sensitive to the metals. Our findings suggest that EPSs are necessary for the protection of bacteria from either Hg (II) or As (III) stress. Previous studies have described a pump in *S. meliloti* that causes efflux of arsenic from cells to surrounding culture medium, thereby protecting them from this type of chemical stress. The presence of heavy metals or metalloids in culture medium had no apparent effect on formation of biofilm, in contrast to previous reports that biofilm formation helps protect various microorganism species from adverse environmental conditions. In co-culture experiments, EPS-producing heavy metal resistant strains exerted a protective effect on AEPS-non-producing, heavy metal-sensitive strains; a phenomenon termed “rescuing” of the non-resistant strain.

## 1. Introduction

The nitrogen-fixing symbiosis is the result of a complex interaction by which a legume plant and a type of bacteria (rhizobia) both obtain nutritional benefit. The bacteria supply the plant with reduced nitrogen from atmospheric sources that are not directly available to the plant; while the bacteria obtain carbon compounds from the plant within the protected root nodule [1]. Specifically *Sinorhizobium meliloti* (*S. meliloti*), under nitrogen limitation conditions, is able to engage in a symbiotic association with the agriculturally significant legume *Medicago sativa* (alfalfa) [2].

*S. meliloti* produces two different EPSs commonly known as EPS I (succinoglycan) and EPS II (galactoglucan) [3], which are both able to promote symbiosis. EPS I, the best-understood symbiotically important EPS, is required for the invasion of alfalfa roots by *S. meliloti* strain Rm1021 [4]. EPS I is a polymer of repeating octasaccharide subunits (seven glucoses and one galactose), bearing succinyl, acetyl, and pyruvyl substituents [5]. On the other hand, EPS II is composed of alternating glucose and galactose residues that are acetylated and pyruvylated, respectively [6]. EPSs are produced in dual forms, having high molecular weight (HMW) and low molecular weight (LMW). The LMW fraction is an active biological form of EPS that is essential for the successful infection of leguminous plants that form indeterminate-type nodules [7]. Under nonstarvation conditions in the laboratory, wild-type *S. meliloti* Rm1021 produces detectable quantities of succinoglycan but does not produce EPS II. This strain, Rm1021, carries an insertional mutation within the *expR* gene [8] that prevents EPS II production under standard culture conditions. The presence of a functional *expR* open reading frame (ORF) on a plasmid or in the genome is sufficient to promote the production of symbiotically active EPS II, e.g., in strain Rm8530, which has an intact, functional *expR* locus and is therefore termed *expR^+^* [9].

Biofilms are commonly defined as bacterial communities in which cells are embedded in a matrix of extracellular polymeric compounds attached to a surface [10,11]. Living in biofilms helps protect bacteria from deleterious conditions and the formation of biofilms appears to be an important factor in the disease cycle of bacterial pathogens in both animals and plants. Bacterial surface components and extracellular compounds such as flagella, lipopolysaccharides, and exopolysaccharides, in combination with environmental and quorum-sensing signals, are crucial for autoaggregation and biofilm development [12].

Rhizobia develop structured biofilms and the specific information about the four major genera: *Mesorhizobium*, *Sinorhizobium*, *Bradyrhizobium*, and *Rhizobium* have been summarized [13]. Particularly for *S. meliloti* Rm1021 strain, attachment to polystyrene and growth as a biofilm depend on the environmental conditions [14], and biotic and abiotic surface colonization is affected by succinoglycan production [15]. *S. meliloti* strain Rm8530, which has a mucoid phenotype, forms a highly structured architectural biofilm, in contrast to the unstructured one formed by non-EPS II producing the strain Rm1021 [16]. The presence of a functional copy of the *expR* regulator gene is necessary for autoaggregation. LMW EPS II, either alone or in combination with the HMW fraction, may function as a polymeric extracellular matrix that agglutinates bacterial cells [17]. In consequence, wild-type *S. meliloti* reference strains carrying nonfunctional *expR* loci fail to autoaggregate and develop a relatively small biomass attached to plastic surfaces and, therefore, a functional EPS II synthetic pathway and its proper regulation are essential for cell-cell interactions and surface attachment of *S. meliloti*. In addition, we found a positive correlation between bacterial autoaggregation and biofilm formation in native *S. meliloti* strains isolated from root nodules of alfalfa [18].

Heavy metal pollution of soil is a significant environmental problem and has a negative impact on human health and agriculture. A variety of microbial mechanisms exist for metal resistance, including physicochemical interactions (adsorption to cell wall and other constituents), efflux, intracellular sequestration and/or extracellular precipitation by the excreted metabolites [19]. Because of this, the interaction between microbial anionic polymers and heavy metals has important ecological and practical implications because it can be useful for removing toxic heavy metals from solutions. Among the molecules able to bind heavy metals are bacterial polysaccharides, linked to the cell surface and excreted to the medium. Although EPSs production in response to heavy metals has been studied in other bacterial species [20,21], few studies on rhizobia have been performed. Among them, may be mentioned the precipitation of metallic cations by the acidic exopolysaccharides from *Bradyrhizobium* strains [22], the capacity of the EPSs of *Rhizobium etli* to bind to metal ions [23], the complexation of Cd^2+^ by the extracellular polymeric substances in *S. meliloti* [24], and the tolerance to acidity and aluminum mediated by EPSs production in *Rhizobium* strains [25].

Microbial biofilms, natural or engineered, could be used to remediate heavy metal pollution by biochemical modification and/or the accumulation of toxic metal ions [26]. The accumulating data suggest that metals, if present in greater concentrations in soils, have substantial deleterious effects on both survivability and nitrogen-fixing efficiency of symbiotic rhizobia [27]. We have shown previously that rhizobial cell surface components such as EPSs, in combination with bacterial functional signals, are essential for the processes of autoaggregation and biofilm formation [13]. In this study, we evaluated the possible involvement of *S. meliloti* EPSs and the consequent biofilm formation on bacterial resistance to heavy metals.

## 2. Results

### 2.1. Bacterial Growth

Different bacterial strains of *S. meliloti* such as Rm8530 wild type (WT) and mutants deficient in the production of EPS I (Rm8530 *exoY*), EPS II (Rm8530 *expA*), and both EPS I and EPS II (Rm8530 *exoYexpA*), were grown in TY medium supplemented with either Hg (II) or As (III) at different concentrations (see Materials and Methods). This was done to ascertain the effect of these toxic metal species in cell growth. Survival of strains was measured through building growth curves and quantification of cfu·mL^−1^ in the different conditions. In general, the variation of concentration of the different metals did not affect the behavior of the strains (data not shown). This demonstrated that, at least for the concentrations tested in the present work, the presence of the metal was a key factor affecting the bacterial growth in a different way from the concentration. According to this evidence, the results presented correspond to the highest concentration of each metal probed, in this case 100 µM of NaAsO_2_ and 20 µM of HgCl_2_ (hereafter referred to as As 100 and Hg 20).

**Figure 1:**
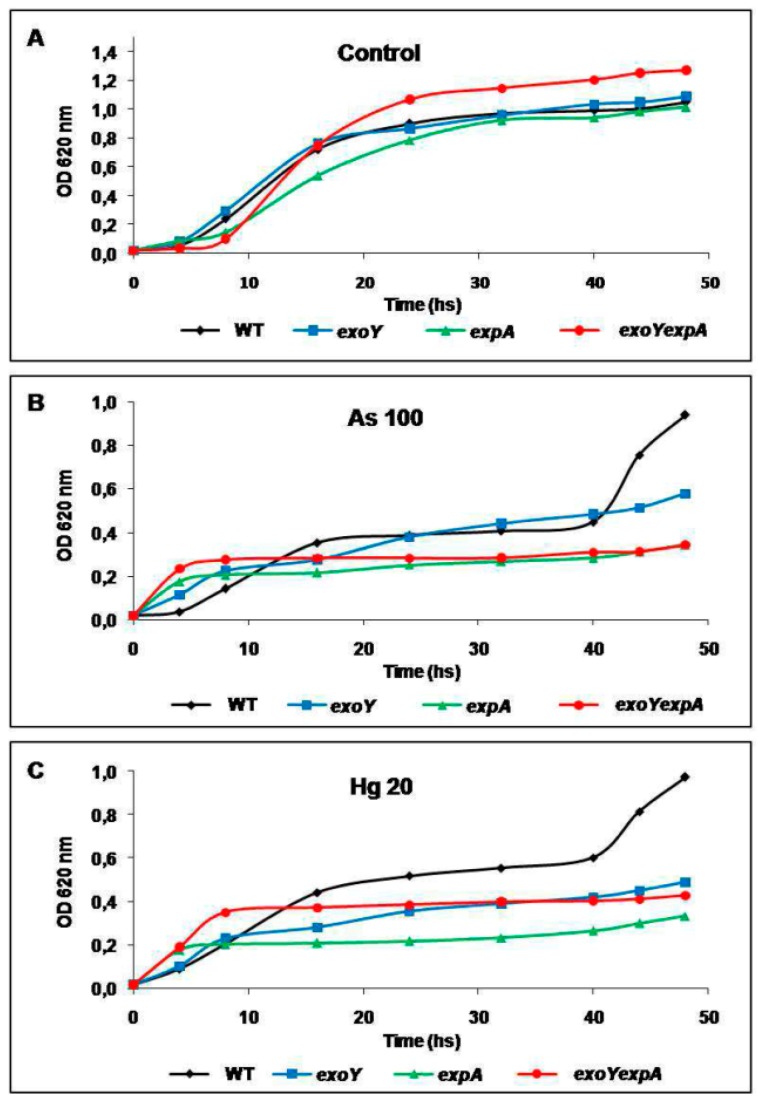
Growth curves of *S. meliloti* strains. Rm8530 wild type (WT) strain and Rm8530 strain mutants in the synthesis of EPS I (*exoY*), EPS II (*expA*), and both EPSs (*exoYexpA*) were grown for 48 h in TY medium without metals added (control) (**A**); and in TY medium supplemented with NaAsO_2_ 100 µM (As 100) (**B**); and HgCl_2_ 20 µM (Hg 20) (**C**). Each point represents the media value of three different assays performed in triplicate. Error bars are omitted for a better visualization of the graphic.

Figure 1 shows the growth curves of the four *S. meliloti* strains evaluated in TY medium without any metal added (control) and in presence of either As 100 or Hg 20. In control conditions, all strains showed typical growth curves with a lag phase of approximately 5–8 h, an exponential phase until approximately 25 h, and a stationary phase since 25–30 h onwards (Figure 1A).

Growth curves obtained in presence of either As or Hg metal (Figure 1B,C), were, in general, similar for each strain tested. The growth curves were characterized by an initial slight increased in absorbance, followed by a growth stopping which conduced to draw a plateau in the curves of the Rm8530 strains in presence of As or Hg (Figure 1B,C). The WT strain was able to recover its growth after 40 h and it reached an optical density (OD) similar to control conditions at the end time of the assay (48 h) in presence of As and a slightly minor in presence of Hg. All mutant strains were incapable of recover their growth in As or Hg as compared to control, and they did not reach an OD superior to 0.4. The Rm8530 *exoY* mutant reached higher absorbance at the end of the assay compared to the other mutants (*expA* and *exoYexpA*). This effect was more marked in As 100 (Figure 1B,C).

In order to validate the results obtained from absorbance measurements, bacterial counts were made at 0, 24 and 48 h. All strains had an initial count (time 0 h) of approximately 1 × 10^2^ colony forming units per mL (cfu·mL^−1^) (data not shown). In all conditions, each strain showed different counts between 24 and 48 h, thus indicating that they were able to grow with time (Table 1). Results of bacterial counts (Table 1) showed a correlation with bacterial growth assessed by measurements of absorbance (Figure 1). Under control conditions, bacterial counts were higher compared to exposure to As or Hg, and WT and *exoY* strains reached counts in the order of 10^9^ cfu·mL^−1^, being slightly superior to mutants in the synthesis of EPS II (*expA* and *exoYexpA*). As shown in Table 1, the metal (As or Hg) reduced the counts of all strains compared to control medium at 24 h. At 48 h, that effect was reverted only for the WT strain, which reached counts of 10^9^ cfu·mL^−1^, while counts of mutant strains were clearly affected by the heavy metal. The EPS II producer *exoY* mutant strain was able to adapt better to either metal, As or Hg compared to the other mutants. It reached bacterial counts of 10^7^ cfu·mL^−1^, whereas *expA* mutant and the double mutant reached bacterial counts of 10^5^ cfu·mL^−1^ and 10^6^ cfu·mL^−1^, respectively. All results of bacterial counting (Table 1) coincide with the behavior of strains in the growth curves obtained through OD measurement (Figure 1).

### 2.2. Bacterial Biofilm Formation

**Figure 2:**
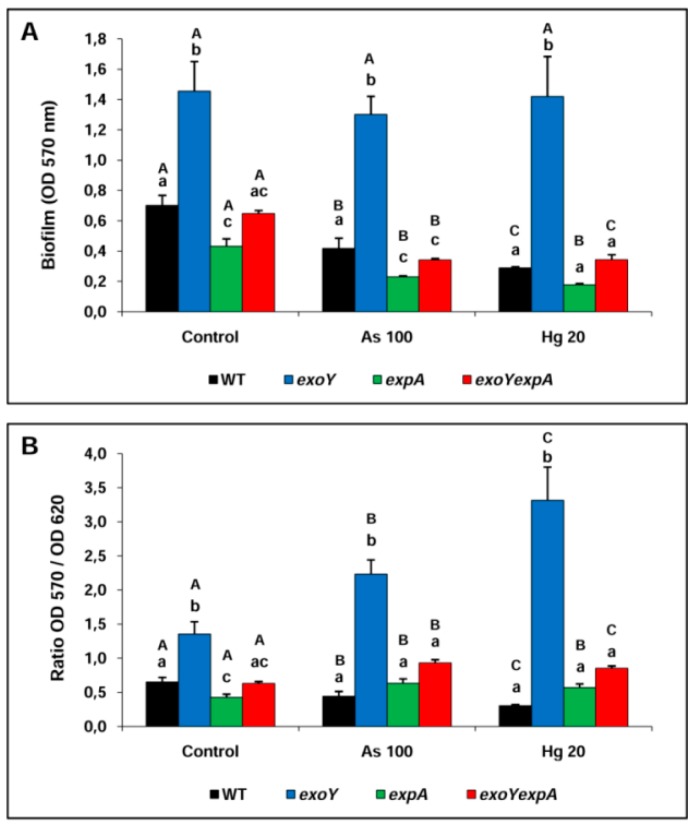
Bacterial biofilm formation (**A**); and relation between biofilm formation and planktonic growth (**B**) of *S. meliloti* strains in control and exposed to heavy metal conditions. Different small letters indicate statistic differences between strains of a same condition according to Fisher’s LSD test (*P* < 0.05). Different capital letters indicate statistic discrepancies between conditions for a given strain according to Fisher’s LSD test (*P* < 0.05).

The ability to form biofilm for different strains belonging to *S. meliloti* derived from Rm8530 cluster was evaluated under exposure to the heavy metal Hg (II) and to the metalloid arsenite (As III). As exposed above for bacterial survival, in this work, the role of EPSs of *S. meliloti* Rm8530 strains and their contribution in the process of biofilm formation as a mechanism for dealing with the toxic effect of As and Hg was evaluated. For this purpose, Rm8530 WT strain and mutants strains in the EPSs synthesis were studied through qualitative (adhesion assay of crystal violet, Figure 2) and quantitative (cfu·mL^−1^ in phases planktonic and sessile, Table 2) methods (see materials and methods section).

In coincidence with previous studies [16,18], Figure 2A shows that the EPS II producer *exoY* strain was the most capable of forming biofilm compared to the fully EPSs producer WT strain and the EPS II deficient strains (*expA* and *exoYexpA*). In contrast to previous studies carried out with different rhizobacteria [28,29] and with extremophile bacteria [30,31] which increased their ability of forming biofilm during metal stressed conditions; the presence of As or Hg in the culture medium reduced the ability of the *S. meliloti* strains of forming biofilm, with exception of the overproducer EPS II *exoY* strain. In this sense, the *exoY* strain was superior in producing biofilm in each condition compared to the rest of the strains and it was not affected by the toxic effect of the metals compared to the control condition.

Figure 2B shows the ratio OD_570_/OD_620_ obtained between sessile cells (cells associated as biofilm) and planktonic cells (free cells in the culture medium) respectively. This ratio is a useful parameter used to identify the dominant lifestyle in a given condition. With exception of WT strain, the Rm8530 mutant strains showed higher values of ratio OD_570_/OD_620_ when they were exposed to As or Hg. According to this ratio, the *exoY* strain was the most capable of adhering to the support in the presence of metals. In such conditions, it is clear that most of the biomass is part of a biofilm compared to free cells. However, these observations were obtained by indirect determinations (OD measurements) and may not definitely correspond to viable cells. Based on this, the cfu·mL^−1^ in each cell fraction was quantified to arrive at more conclusive findings (Table 2). In this sense, comparisons among cells counts from bacterial cells free in the culture medium (planktonic phase) and bacterial cells free released from biofilm adhered to glass (sessile phase) were carried out.

With the exception of WT strain, the planktonic cell counts of all strains studied were reduced when the strains were exposed to heavy metals compared to control medium. The EPS II non-producer strains (*expA* and *exoYexpA*) showed very low sessile count in all conditions evaluated (3.35 × 10^3^ and 5.25 × 10^2^ cfu·mL^−1^ for control, 2.04 × 10^2^ and 2.14 × 10^2^ cfu·mL^−1^ for As 100, and 1.84 × 10^2^ and 2.26 × 10^2^ cfu·mL^−1^ for Hg 20, respectively), demonstrating the inability of these strains for forming biofilm. In contraposition to these results, the sessile cfu·mL^−1^ of the EPS II producer WT and *exoY* strains were higher in all conditions tested, although the presence of As or Hg caused reductions in the cell count in one (~10^8^ cfu·mL^−1^, *exoY*) or two (~10^7^ cfu·mL^−1^, WT) orders of magnitude compared to the control condition (Table 2). Particularly and coincidentally with the qualitative results (Figure 2), the *exoY* mutant showed a higher sessile count in all conditions as compared to the WT strain. Most interesting in reflecting the tendency of a particular strain to choose (in biofilm) is the efficiency percent (Table 2, Ef%). It refers to the fraction of sessile cells in respect of total cells in a given condition. On each condition tested, the *exoY* strain showed the higest Ef% followed by the WT strain; whereas the *expA* and the double mutant showed very little efficiency for forming biofilm. The WT strain reveal a reduced Ef% in presence of heavy metals compared to control condition. The *exoY* strain increased their Ef% when it was exposed to heavy metals. Despite their low Ef%, the *expA* and the double mutant showed a slight increase in this parameter under exposure to As or Hg.

### 2.3. Bacterial Co-Culture

Results of biofilm formation showed above highlight the role of production of EPSs as a possible resistance mechanism to metal stress exposure. In order to check that evidence and to further establish the role of EPSs of *S. meliloti* in resistance to As and/or Hg, a couple of bacterial co-culture assays were performed between a fully EPS producer strain (WT) or an EPS II producer strain (*exoY*) and a non EPS producer strain (*exoYexpA*). Qualitative (growth through OD_620_ nm measurements and biofilm formation through crystal violet technique and OD_570_ nm measurement) and quantitative (bacterial count of planktonic and sessile cells) parameters were determined in individual cultures and in co-cultures of selected strains. The phenomena of rescue, the recovery of a defective strain in co-culture with other strains with respect to the individual culture, were assessed in planktonic and in sessile phases.

#### 2.3.1. Rm8530 WT–Rm8530 Exoyexpa Co-Culture

The behavior of the co-culture of the fully EPSs producer Rm8530 WT strain and the non EPSs producer double mutant Rm8530 *exoYexpA* strain was studied.

**Figure 3:**
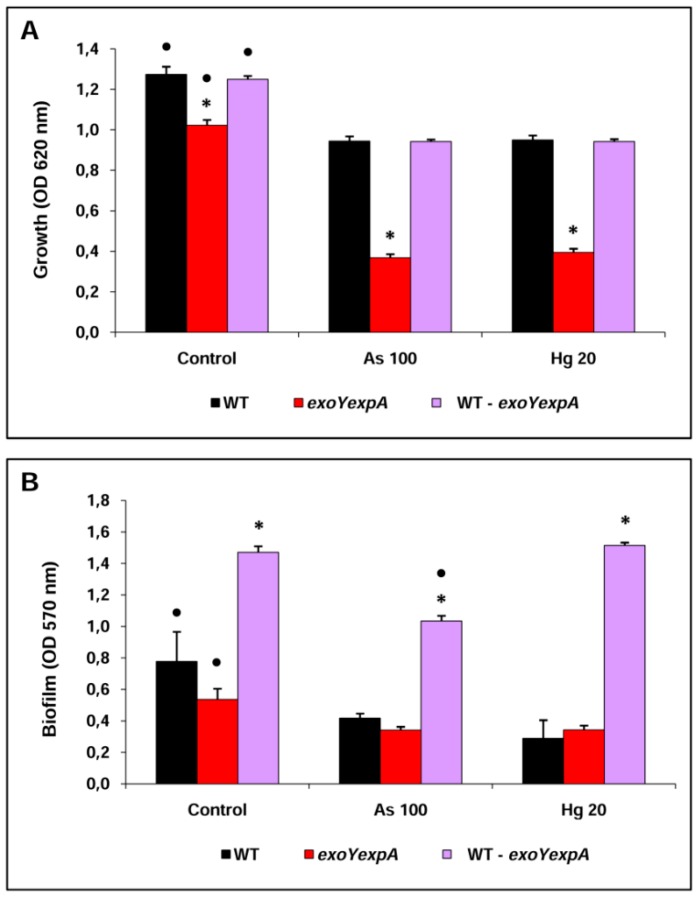
Bacterial growth (**A**) and biofilm formation (**B**) of individual and co-culture *S. meliloti* strains in control and exposed to heavy metal conditions. Bars marked with * indicate statistic differences among individual or co-culture strains for the same condition according to Fisher’s LSD test (*P* < 0.05). Bars marked with • indicate statistic differences between conditions for a given strain according to Fisher’s LSD test (*P* < 0.05).

The growth (OD_620_ nm) of the individual culture of the Rm8530 *exoYexpA* strain was lower in each condition (control, As 100 and Hg 20) compared to both, the individual culture of the Rm8530 WT strain and the co-culture (Figure 3A). The presence of either As or Hg, in the culture medium, reduced the growth of individual cultures or co-culture as compared to control condition. The effect was more marked in the individual culture of the double mutant growing in metals, whereas the co-culture grew in a similar manner to the individual culture of the WT strain (Figure 3A).

The assay of biofilm formation (OD_570_ nm) showed that, for each condition (control, As 100 and Hg 20), the ability of strains to form biofilm in co-culture was higher as compared to individual cultures (Figure 3B). The exposure to As or Hg reduced the biofilm formation of individual cultures as compared to control condition, whereas the co-culture had a lower ability to form biofilm under exposure to As compared to the co-cultures in control or Hg conditions (Figure 3B).

The bacterial count of planktonic cells showed that, with exception to the individual culture of the WT strain, the cfu·mL^−1^ were lower for the individual culture of the double mutant and lower for each strain in the co-culture in presence of metal as compared to control condition without metal (Table 3). An interesting datum is that the composition of the co-culture was formed predominantly by the WT strain in control and Hg conditions. However, the composition of the co-culture exposed to As was constituted equally by each strain. Accordingly, the rescue index of the double mutant strain in co-culture with respect to the individual culture was approximately 16 times higher in the As 100 condition (Table 3), thus suggesting that the WT strain provides a rescue mechanism for the double mutant strain in presence of As.

Results obtained for the cells established in the biofilm (sessile cells) of co-culture are very interesting. The bacterial count of WT strain sessile cells from individual culture were lower (~10^7^ cfu·mL^−1^) when exposed to metals compared to control condition (~10^9^ cfu·mL^−1^), whereas the bacteria from the individual culture of the double mutant strain was practically incapable to form biofilm independently of the condition, yielding an insignificant sessile cell count (~10^2^ cfu·mL^−1^) (Table 3). Curiously, the sessile cell counts resulting from the co-culture showed a biofilm formed by a large cfu·mL^−1^ of both WT and *exoYexpA* strains, in all conditions studied. The WT strain in co-culture showed a higher sessile cell count compared to the individual culture and this result was more marked in the conditions of exposure to As or Hg. The double mutant in co-culture becomes in a strain able to form part of the biofilm, it increased their sessile cell count from ~10^2^ cfu·mL^−1^ (in each condition) in individual culture to 10^7^ (As 100), 10^8^ (Hg 20) or 10^9^ cfu·mL^−1^ (control) when growing with the WT strain. Although the co-culture of biofilm composition was predominantly constituted by the WT strain ranging from ~99% in Hg to ~79% in control, the rescue index of sessile cells of the double mutant strain in co-culture respect to individual culture was amazingly high. In this sense, the *exoYexpA* strain was approximately ~10^6^ (control) and ~10^5^ times recovered in the biofilm obtained from co-culture conditions (Table 3).

#### 2.3.2. Rm8530 Exoy–Rm8530 Exoyexpa Co-Culture

The co-culture between the EPS II producer Rm8530 *exoY* strain and the non EPSs producer double mutant Rm8530 *exoYexpA* strain was studied.

**Figure 4:**
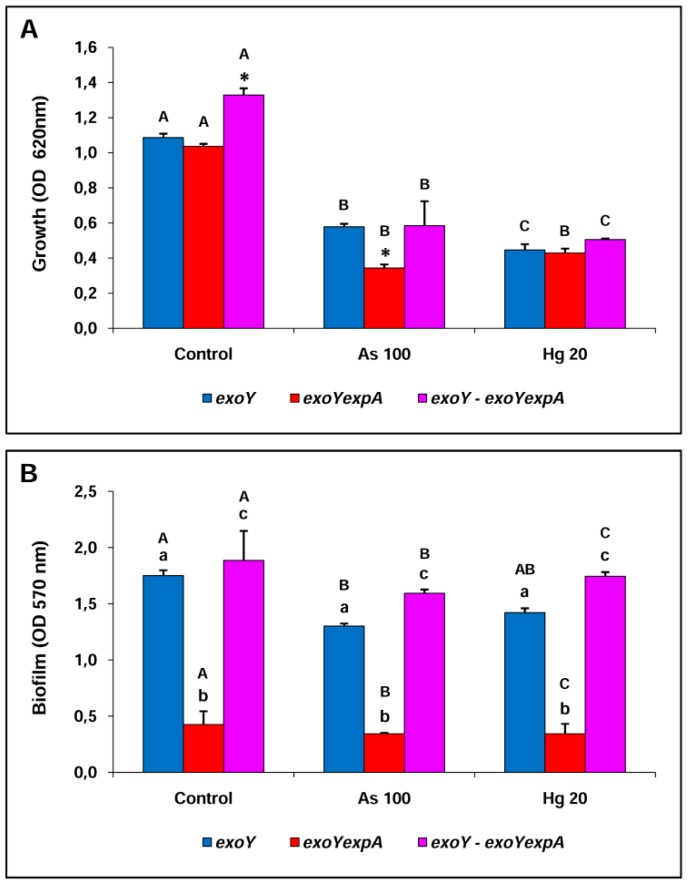
Bacterial growth (**A**) and biofilm formation (**B**) of individual and co-culture *S. meliloti* strains in control and exposed to metal conditions. (**A**) Bars marked with * indicate statistic differences among individual or co-culture strains for the same condition, whereas different capital letters indicate statistic differences between conditions for a given strain according to Fisher’s LSD test (*P* < 0.05); (**B**) Different small letters indicate statistic differences among individual or co-culture strains for the same condition and different capital letters indicate statistic differences between conditions for a given strain according to Fisher’s LSD test (*P* < 0.05).

As was previously determined (Figure 3A), the growth (OD_620_ nm) of the individual culture of the Rm8530 *exoYexpA* strain was lower when exposed to metal condition (As 100 or Hg 20) compared to control condition. A similar effect was determined for the individual culture of Rm8530 *exoY* mutant as well as for the co-culture (Figure 4A).

Coincidentally with the results shown above (Figure 2A and Figure 3B), the double mutant was severely affected on its ability to form biofilm in all conditions evaluated, being the effect emphasized under metal exposure (Figure 4B). For each condition, the assay of adhesion to glass support showed that the co-culture was superior in their ability to form biofilm compared to individual cultures, although the values obtained were similar to the individual culture of Rm8530 *exoY* mutant (Figure 4B). These results support the possibility of a rescue mechanism of the *exoY* strain for the double mutant strain.

Table 4 shows the bacterial count for planktonic and sessile cells of individual and co-culture of Rm8530 *exoY* strain and Rm8530 *exoYexpA* strain. The bacterial planktonic count showed that the cfu·mL^−1^ were lower in the individual culture of each strain in presence of metal compared to control condition. However, the cell number was notably increased in the planktonic phase of each condition when the strains were growing in co-culture. This effect was more marked in presence of metal and it was also reflected in the rescue index which was higher in As (~423) with respect to Hg (~110) and control (~8). The composition of the co-culture in the planktonic phase was of approximately 60% *exoY* strain/40% double mutant strain for control and Hg conditions and slightly imbalanced in favor of *exoY* strain in As condition (75/25) (Table 4).

The bacterial count in the sessile phase Rm8530 *exoY–*Rm8530 *exoYexpA* co-culture was similar to those obtained for Rm8530 WT*–*Rm8530 *exoYexpA* co-culture. In this sense, it was clearly determined that the EPS II producer *exoY* strain was able to rescue to the EPSs non-producer *expAexoY* strain. The sessile cell count of the *exoY* strain from individual culture was slightly lower (~10^8^ cfu·mL^−1^) when exposed to metals compared to control condition (~10^9^ cfu·mL^−1^). On the other hand, and independently of the condition, the population of the sessile bacteria from individual culture of the double mutant strain was very low (~10^2^ cfu·mL^−1^), a fact that is coincident with an incapability to form biofilm (Table 4). The *exoY* strain in co-culture showed similar sessile cell counts compared to the individual culture. However, the double mutant was greatly favored from living in consortia with the *exoY* strain, reaching higher cell numbers in the mixed biofilm (~10^8^ cfu·mL^−1^ and 10^9^ cfu·mL^−1^). Similar to what was describe above (Table 3), the rescue indexes obtained for each condition highlight the fact that in co-culture, the double mutant becomes a strain able to form part of the biofilm showing rescues of approximately 10^6^ (control and Hg) and 10^5^ (As) times in the mixed biofilm with *exoY* strain (Table 4). Composition of mixed biofilm was characterized by the major proportion of the *exoY* strain in all conditions; however, the exposure to metals equalized the biofilm composition.

## 3. Discussion

As a whole, and despite the difference between the chemical nature of the toxic elements evaluated, *i.e.*, As as a metalloid and Hg as a metal of transition, substantial differences were not found in the distinct essays of exposure of *S. meliloti* to these elements. It suggests that *S. meliloti* is affected in a similar way by As and Hg, and that possibly this bacteria can have a metabolic/physiologic mechanism to deal with these toxic elements.

A well-known and efficient mechanism of mercury resistance among Gram-positive and Gram-negative microorganisms (including *S. meliloti*) [32] involves mercury reduction encoded by the microbial *mer* operon located on mobile genetic elements [33,34].

Resistance to As in *S. meliloti* depends on *ars* operon which consists of *arsR* (transcriptional regulator), *arsC* (arsenate reductase) and *aqpS* (aquaglyceroporin). The AqpS is the only protein of the *S. meliloti ars* operon that facilitates transport of arsenite and flows out of the cell [35]. Moreover, a protein codified by *arsH* gene has been described as a nicotinamide adenine dinucleotide phosphate (NADPH): flavin mononucleotide (FMN) oxide-reductase that forms H_2_O_2_ in *S. meliloti* exposed to As [36].

Despite the involvement of all that machinery of metal resistance, we demonstrate, in this work, the impact of the *S. meliloti* EPSs in conferring metal resistance in this rhizobia.

Results obtained from growth curves (Figure 1) and bacterial counts (Table 1) clearly suggest that the EPSs of *S. meliloti* can have a protective role against the exposure to toxic metals. In this sense, in presence of either As or Hg, the Rm8530 WT strain was able to reach OD or cfu·mL^−1^ similar to control condition without metal, whereas mutants defective on the synthesis of EPSs were not capable, in presence of metals, of achieving the growth parameters reached under control conditions. Coincidentally, previous works had reported the diminished growth rate of several soil bacterial strains growing in the presence of heavy metals or metalloids [28,29]. It was also determined that the WT strain grew slowly when As or Hg were in the culture medium. However, it was able to recover at the end of the assay. This phenomena of long adaptation to the presence of the metals and of being, later on, able to achieve a growth similar to control without metal suggests that possibly the production of EPSs by the WTs strain could sequester the toxic metal, giving to the bacteria the time required for adaptation thus driving to the physiological or metabolic changes necessary for eliminating the toxic effect of the metal, *i.e.*, expression of enzymes and transporters for pumping out the metal or metal-binding proteins [35,37,38,39,40]. This effect was not observed in the *S. meliloti* mutant strains suggesting that, despite having the complete genetic/metabolic tools for metal resistance, the absence of fully EPSs production for trapping metals drives to metal toxicity before other resistance mechanisms can be expressed. Nevertheless, the overproducing EPS II/non-producing EPSI Rm8530 *exoY* strain showed a better survival (OD or cfu·mL^−1^) during the exposure to As or Hg compared to the non-producing EPS II/producing of EPS I Rm8530 *expA* strain or the non-producing EPSs Rm8530 *exoYexpA* strain. These results support that the EPS II would be more relevant that the EPS I in dealing with the toxicity of heavy metals/metalloids.

Although it is well known that bacteria produce more biofilm during exposure to metal-stressed conditions, results from the present work for different strains of *S. meliloti* are discordant. In this sense, biofilm ability was reduced or almost not modified by the presence of metals in the culture medium (Figure 2) for either, most of *S. meliloti* strains or Rm8530 *exoY* strain, respectively. Biofilm is an array of bacterial cells imbibed in an extracellular polymeric substance, responsible for protecting bacterial cells from toxic metals by binding it, retarding their diffusion within the biofilm [41]. The biofilm structure can be associated with a protective mechanism which allows bacteria to survive and thrive in environments containing high concentrations of heavy metals or metalloids [42]. In this sense, biofilm induction has been described for *Herminiimonas arsenicoxydans* [30] and *Thiomonas arsenitoxydans* [31] exposed to high As (III) concentrations. Development of this capacity allows bacteria to attach on environmental surfaces and thereby facilitates their physiological and metabolic adaptation to successfully survive in metal polluted environments [28].

Biofilm formation of *S. meliloti* exposed to metal stressed conditions is quite conclusive regarding the role of EPSs in this bacteria. In this sense, the function of the EPS II as the key factor in the process of biofilm development of *S. meliloti* [16] was also crucial for the resistance to toxic metals of EPS II producer strains. Particularly, the EPS II overproducer Rm8530 *exoY* was the strain with the highest ability to form biofilm independently of the condition. Their ratio biofilm/growth (OD_570_/OD_620_) increased as a consequence of exposure to As with respect to control and it was even higher when exposed to Hg. These observations had a correlation with the sessile cell count and the Ef% which increased from ~25% in control medium to 83% and 91% in presence of As or Hg, respectively. It can be concluded that, at least for the EPS II producer strains, the exposure of *S. meliloti* to toxic metals drives the bacterial cells to the formation of a population associated under a biofilm lifestyle, probably as a strategy to improve survival in this situation of stress. Although the WT strain (which produces both, EPS I and EPS II compounds) was better biofilm producer in all conditions compared to *expA* and *exoYexpA* strains, it showed reduced parameters of biofilm formation in all conditions as compared to the *exoY* strain. This fact can be explained by the overproduction of EPS II of the *exoY* strain (despite the fact that EPS II has not been measured, phenotype of *exoY* strain growing on plates of culture is very mucoid and polimer produced drips, data are not shown) or in the interference that EPS I can have in the development of biofilm in the WT strain. Coincidentally, the role of EPSs production as a mechanism for metal resistance has been described for other rhizobia, rhizobacteria and extremophiles. EPSs production in *Bradyrhizobium japonicum* E109 and *Azospirillum brasilense* Az39 increased significantly under 25 μM and 500 μM of As (III) exposure, respectively [29]. A proteomic study carried out in *H. arsenicoxydans* showed that expression of proteins was required not only for arsenic detoxification or stress response but it was also involved in motility, exopolysaccharide synthesis, phosphate import or energetic metabolism, thus suggesting that exposure to toxic metals drives to global changes in bacterial physiology [43]. Moreover, supporting findings of the present work, besides genes involved in arsenic resistance and metabolism [44,45], show that *H. arsenicoxydans* has acquired the property of producing a thick capsule of exopolysaccharides, which has been shown to scavenge arsenic as granules [42]. It was also demonstrated that the exopolymeric substance of an environmental mature biofilm contained higher concentrations of metals and carbohydrates than exopolymeric substances from initial/immature biofilm, thus showing that biochemical composition of biofilms is dependent on maturity and is controlled by the microbial communities, as well as by the local geochemical environment [46].

Most biofilms in nature consist of multispecies of microbial consortia [47,48,49]. Several studies showed the inability of certain bacteria to form biofilms individually but in synergism with other bacterial strains, such structures could be formed [50,51]. Biofilms of two bacterial strains are simplified models to verify microbial interactions such as synergism, antagonism and study of the determinants of such interactions (EPSs, quorum sensing mechanism, motility, *etc.*) [52]. Although reports are not abundant, study of the formation of mixed biofilms between different species has acquired great interest because of their possible applications like in the bioremediation of contaminated soils [51,53]. Despite these previous reports, little is known about the dual biofilm of bacteria exposed to metals.

In this work, we show the role of the EPSs of *S. meliloti* in process of metal tolerance and bacterial survival through the study of co-cultures between EPSs producer strains (WT or *exoY*) and a non EPSs producer strain (*exoYexpA*). The most relevant impact of the results obtained in the co-culture studies were those acquired from the cell count in planktonic and sessile phases. Particularly, the most surprising finding was the ability of the EPSs producer strains for rescuing the EPSs non-producer strain, so increasing their survival in planktonic phase and allowing their formation as a mixed biofilm in sessile phase. It was previously shown that mercury-reducing biofilms from packed-bed bioreactors, treating nonsterile industrial effluents, change from initial monospecies biofilms of *Pseudomonas putida* to a mixed biofilm consisting of invading additional strains, two of which were not mercury resistant, demonstrating phenomena of rescuing in environmental conditions [54,55]. Here, we can describe two possible phenomena for the results obtained of cell count found in the mixed biofilm in the co-culture WT-*exoYexpA* (Table 3): (i) one is a cooperative effect in presence of metals between the WT and the *exoYexpA* mutant based on the higher cfu·mL^−1^ compared to individual cultures; and/or (ii) the other is a rescue process in all conditions of the WT to the double mutant giving the amazing cfu·mL^−1^ of double mutant sessile cells compared to its individual culture. The major planktonic cell count and rescue indexes for the co-culture *exoY–exoYexpA,* in respect of individual cultures (Table 4), can hold the idea of the protective effect of EPS II produced by the *exoY* strain, so conferring resistance to metals and rescue of the EPSs non-producer strain. This was less evident for the co-culture WT*–exoYexpA* probably due to the minor production of EPS II for synthesis of the EPS I in the WT strain (Table 3). It also holds the idea that a microorganism (*i.e.*, EPS II non-producer strains) is not be capable of forming biofilm under increased concentrations of toxic metals. However, once the structure is formed (*i.e.*, by an EPS II producer strain), it may protect the enclosed cells independently of the strains trapped, even from higher concentration of the toxic compound. Global results presented in this work clearly determined that the EPS II producer strains (WT and *exoY*) were able to rescue the EPSs non-producer strain (*exoYexpA*), supporting the idea that EPSs provides bacterial protection against metal toxicity [56,57]. Results demonstrated that EPS II producer strains provide the propitious scenario for the establishment in biofilm of the EPSs non-producer strain, so improving/allowing their survival, even under exposure to toxic metals.

Several bacteria have been described as metal resistant, being able to grow in metal polluted environments. Therefore, the utilization of microorganisms in contaminated environments represents a promising solution for metal remediation [58,59,60,61,62,63,64]. Findings of the present work imply the potential usage of *S. meliloti* in improvement growth of alfalfa plants in metal/metalloid contaminated soil, providing evidence of the key role of EPSs as a probable molecular determinant of bacterial survival in metal stressed soils and consequently allowing the development of symbiosis in such polluted environments.

## 4. Materials and Methods

### 4.1. Bacterial Growth Conditions

#### 4.1.1. Bacterial Strains, Culture Media, and Growth Conditions

*S. meliloti* strains were grown at 30 °C in TY medium [65] on a rotary shaker (Model SI4-2 Shel Lab, 12 mm orbit, Sheldon Manufacturing Inc., Cornelius, OR, USA) at 200 rpm, as described previously [16]. Antibiotics were used at the following final concentrations: streptomycin (500 μg/mL), neomycin (200 μg/mL), gentamicin (40 μg/mL), tetracycline (10 μg/mL) and cycloheximide (25 μg/mL). All antimicrobials are purchased from Sigma-Aldrich (St. Louis, MO, USA). Strains used are listed in Table 5.

#### 4.1.2. Stock Solutions of Arsenic and Mercury

Stock solutions of NaAsO_2_ (Sigma-Aldrich, St. Louis, MO, USA) and HgCl_2_ (Cicarelli, San Lorenzo, Argentina)10 mM were prepared to achieve final concentrations in the culture media of 25 µM, 50 µM, 100 µM of NaAsO_2_ and 2.5 µM, 5 µM, 10 µM, 20 µM of HgCl_2_. The salt solutions were sterilized by filtration with filter MC-PES-02S (JetBiofil^®^ Syringe Filter, Guangzhou, China) with a pore size of 0.22 μm and preserved at 4 °C. These solutions were used at the indicated concentration to supplement the media in the growth, biofilm formation and co-culture assays.

#### 4.1.3. Growth Response to Metals

Bacterial growth (as above, Section 4.1.1) was assessed by turbidimetry achieved by measuring absorbance at 620 nm. *S. meliloti* was grown in liquid TY control medium without metal and TY medium supplemented with either As or Hg. In all cases, cultures started with an initial OD_620_ nm of 0.01, and adequate aliquots were taken every 4 h in order to determine growth by turbidimetry in a microplate reader (Multiskan MS Primary EIA V 1.8.9, Waltham, MA, USA). After 48 h, data were employed to construct growth curves.

The number of viable cells was determined using the microdroplet technique [66]. It consisted of performing serial dilutions of each sample and sowing on Petri dishes (ExtraGene, Taichung, Taiwan) containing sterile solid medium (TY). Each plate was divided in four fields and two drops (20 µL) of each dilution were placed in each field. The plates were incubated at 28 °C during 48 h, the colonies were counted and the number of colony forming units per mL (cfu·mL^−1^) was calculated.

### 4.2. Biofilm Formation Assay

The biofilm formation was determined macroscopically using a quantitative assay in glass tube (Deltalab, Barcelona, Spain) (10 by 70 mm) in which the biofilm was stained with crystal violet (CV) (Anedra, San Fernando, Argentina) in accordance with the methodology previously described [67]. First, bacterial strains (Rm8530 WT, Rm8530 *exoY*, Rm8530 *expA*, Rm8530 *exoYexpA*) were grown in liquid TY medium with corresponding antibiotics and these cultures were subsequently diluted with sterile physiological saline solution (OD_620_ nm = 1). An aliquot (8 µL) of this bacterial culture dilution was added to glass tubes containing 792 µL of TY culture medium. The As and Hg solutions were added in order to reach the concentrations mentioned above. Immediately after inoculation, the tubes were covered with plastic caps to prevent evaporation and contamination and were incubated on a rotator shaker (150 rpm) at 30 °C for 48 h. Then, the contents of each tube were gently homogenized and bacterial growth was determined by measurement of the absorbance at 620 nm on a microplate reader. Planktonic cells from each tube were then removed with an automatic pipette (Gilson, Villiers-le-Bel, France) and the tubes were washed three times with sterile physiological saline solution (NaCl 0.85%, Cicarelli, San Lorenzo, Argentina). Then, they were emptied and stained with crystal violet 0.1% for 15 min. The dye was carefully retired and tubes were rinsed three times with distilled water. The CV retained in the sessile biomass was solubilized by adding 1 mL of ethyl alcohol (96%) (Porta, Buenos Aires, Argentina). Finally, the OD (570 nm) of solubilized CV was measured with a MicroELISA Auto Reader.

Moreover, the number of viable cells in the biofilm was determined. For this, the culture medium containing planktonic cells from each tube was removed with an automatic pipette. Then, the tubes containing cells adhered to the glass surface (sessile phase, biofilm) were carefully washed four times with sterile saline solution. To confirm that washing was adequate and that no living cells were left, viable cell counts were performed in the last washing step and no cfu were detected. After that, 1 mL of saline solution and 3 sterilized glass beads were added to the washed tubes which were vortexed long enough as to break all the biofilm formed and as to obtain sessile cells uniformly resuspended. Finally, 10-fold serial dilutions were made and the number of sessile cells was determined using the microdoplet technique.

### 4.3. Co-Culture Assays

*S. meliloti* EPS-producing strain was assayed for the capacity to exert a protective effect on EPS-non-producing strain in the presence of metals. To perform these experiments, commonly termed rescue assays, bacterial strains were grown in TY medium supplemented with appropriate antibiotics and incubated 48 h at 28 °C on a shaker at 150 rpm. These cultures were diluted with sterile physiological saline solution to achieve OD (620 nm) = 1. Subsequently, 3 mL of culture medium (TY) were placed on a glass tube with streptomycin 500 µg/mL and 30 µL of each diluted strain culture were added. In presence of the adequate metal concentrations, the tubes were incubated during 48 h at 28 °C under continuous shaking at 150 rpm. Then, determinations of growth and biofilm formation were carried out as described above. A group of tubes were employed to determine the bacterial number expressed as cfu·mL^−1^ in the planktonic and sessile phase by plating the appropriate dilutions in TY plates containing selective antibiotics to differentiate between the strains.

### 4.4. Statistical Analysis

The assays were performed in triplicate and repeated three times. The data were subjected to a one-way analysis of variance (ANOVA), followed by a comparison of multiple treatment levels with the control by using *post hoc* Fisher’s least-significant difference (LSD) test. All statistical analyses were performed using Infostat, version 1.0 (Group Infostat Universidad Nacional de Córdoba, Córdoba, Argentina).

## 5. Conclusions

Several microbial mechanisms exist for metal resistance, including physicochemical interactions, efflux, intracellular sequestration and extracellular precipitation by the excreted polymeric compounds. All the evidence presented here clearly indicates that synthesis of EPS II would be used for *S. meliloti* as a mechanism of resistance to exposure to toxic metals, probably through different effects such as trapping the metal outside the cells and/or formation of biofilm. Both are possible successful mechanisms for reducing metal toxicity in the environment, improving symbiosis development and growth of legume plant in metal polluted soils.

## Figures and Tables

**Table 1:** Bacterial counts of *S. meliloti* strains exposed to metals.

Strain	Condition					
	Control		As 100		Hg 20	
	24 h	48 h	24 h	48 h	24 h	48 h
Rm 8530 WT	3.83 × 10^7^aA	7.06 × 10^9^aA*	1.71 × 10^5^aB	3.49 × 10^9^aA*	2.77 × 10^5^aB	1.23 × 10^9^aA*
Rm 8530 *exoY*	4.97 × 10^7^aA	6.16 × 10^9^aA*	2.94 × 10^5^aB	1.09 × 10^7^bB*	2.95 × 10^5^aB	2.56 × 10^7^bB*
Rm 8530 *expA*	1.19 × 10^6^aA	1.98 × 10^7^bA	2.00 × 10^4^aB	2.98 × 10^5^cB	1.60 × 10^4^aB	2.70 × 10^5^cB
Rm8530 *exoYexpA*	2.93 × 10^6^aA	4.75 × 10^8^aA*	1.17 × 10^4^aB	2.08 × 10^6^bcB*	1.90 × 10^4^aB	2.04 × 10^6^bcB*

Results are the media value of three different assays performed in triplicate and they are expressed as cfu·mL^−1^. Values of standard deviation are omitted to simplify the display and formatting of the table. According to Fisher’s LSD test (*P* < 0.05), different small letters indicate statistic differences between strains; different capital letters indicate statistic discrepancies between conditions for a given strain; and * indicates statistic differences for a strain in the same condition at different times (24 h and 48 h).

**Table 2:** Planktonic and sessile cell count of *S. meliloti* strains on TY medium with different metals.

Strain	Condition								
	Control			As 100			Hg 20		
	PC	SC	Ef (%)	PC	SC	Ef (%)	PC	SC	Ef (%)
Rm 8530 WT	7.46 × 10^9^	1.10 × 10^9^	12.85	2.96 × 10^9^	6.33 × 10^7^	2.09	1.45 × 10^9^	4.35 × 10^7^	2.91
Rm 8530 *exoY*	6.90 × 10^9^	2.37 × 10^9^	25.57	4.34 × 10^7^	2.16 × 10^8^	83.27	3.01 × 10^7^	3.05 × 10^8^	91.02
Rm 8530 *expA*	2.14 × 10^9^	3.35 × 10^3^	1.56 × 10^−4^	1.92 × 10^5^	2.04 × 10^2^	0.11	1.28 × 10^5^	1.84 × 10^2^	0.14
Rm8530 *exoYexpA*	5.56 × 10^8^	5.25 × 10^2^	9.44 × 10^−5^	2.34 × 10^6^	2.14 × 10^2^	0.091	1.44 × 10^6^	2.26 × 10^2^	0.016

Mean values of three different assays performed in triplicate per treatment correspond to cfu·mL^−1^ after 48 h of growth on TY medium (control), TY medium with NaAsO_2_ 100 µM (As 100) and TY medium with HgCl_2_ 20 µM (Hg 20). Values of standard deviation are omitted to simplify the display and formatting of the table. A two-log difference in bacterial count involves statistically significant differences from each other according to Fisher’s LSD test (*P* < 0.05). “Ef (%)” refers to the fraction of sessile cells in respect of total cells, denoting the tendency of a particular strain to choose for a biofilm lifestyle in a given condition. PC: Planktonic cells count. SC: Sessile cells count. Ef (%): (SC × 100)/TC. TC: Total count (PC + SC).

**Table 3:** Planktonic and sessile cell count of individual and co-culture *S. meliloti* WT and *exoYexpA* strains.

***S. meliloti* WT and *exoYexpA* Strains**	**Planktonic Cells**						
	**Bacterial Count**				**Rescue Parameters**		
	**Individual Culture**		**Co-Culture**		**Co-Culture Composition (%)**		Rescue Index *
**Condition**	**WT**	***exoYexpA***	**WT**	***exoYexpA***	**WT**	***exoYexpA***	
Control	7.98 × 10^9^	4.02 × 10^8^	6.18 × 10^9^	8.01 × 10^8^	88.55	11.45	1.99
As 100	3.49 × 10^9^	2.18 × 10^6^	4.64 × 10^7^	3.52 × 10^7^	56.86	43.14	16.15
Hg 20	1.23 × 10^9^	2.01 × 10^6^	2.14 × 10^7^	1.97 × 10^6^	91.57	8.43	0.98
***S. meliloti* WT and *exoYexpA* Strains**	**Sessile Cells**						
	**Bacterial Count**				**Rescue Parameters**		
	**Individual Culture**		**Co-Culture**		**Co-Culture Composition (%) ^#^**		Rescue Index *
**Condition**	**WT**	***exoYexpA***	**WT**	***exoYexpA***	**WT**	***exoYexpA***	
Control	2.82 × 10^9^	2.99 × 10^2^	9.15 × 10^9^	2.46 × 10^9^	78.81	21.19	8.23 × 10^6^
As 100	6.59 × 10^7^	2.35 × 10^2^	4.15 × 10^8^	8.25 × 10^7^	83.42	16.58	3.51 × 10^5^
Hg 20	4.05 × 10^7^	2.12 × 10^2^	9.03 × 10^9^	1.04 × 10^8^	98.86	1.14	4.91 × 10^5^

Mean values of three different assays performed in triplicate per treatment correspond to cfu·mL^−1^ of planktonic or sessile cells after 48 h of growth on TY medium (control), TY medium with NaAsO_2_ 100 µM (As 100) and TY medium with HgCl_2_ 20 µM (Hg 20). Values of standard deviation are omitted to simplify the display and formatting of the table. A two-log difference in bacterial count involves statistic significant differences from each other according to Fisher’s LSD test (*P* < 0.05). **^#^** cfu·mL^−1^ of WT or *exoYexpA* × 100/TC. TC: Total count (cfu·mL^−1^ WT + cfu·mL^−1^ *exoYexpA*). * cfu·mL^−1^ *exoYexpA* strain in co-culture/cfu·mL^−1^ *exoYexpA* in individual culture.

**Table 4:** Planktonic and sessile cell count of individual and co-culture of *S. meliloti* *exoY* and *exoYexpA* strains.

***S. melilotiexoY* and *exoYexpA* Strains**	**Planktonic Cells**						
	**Bacterial Count**				**Rescue Parameters**		
	**Individual Culture**		**Co-Culture**		**Co-Culture Composition (%)**		Rescue Index *
**Condition**	***exoY***	***exoYexpA***	***exoY***	***exoYexpA***	***exoY***	***exoYexpA***	
Control	6.24 × 10^9^	2.69 × 10^8^	3.22 × 10^9^	2.13 × 10^9^	60.19	39.81	7.92
As 100	5.18 × 10^7^	3.17 × 10^6^	3.92 × 10^9^	1.34 × 10^9^	74.52	25.48	422.71
Hg 20	2.51 × 10^7^	1.92 × 10^6^	3.04 × 10^8^	2.11 × 10^8^	59.03	40.97	109.89
***S. melilotiexoY* and *exoYexpA* Strains**	**Sessile Cells**						
	**Bacterial Count**				**Rescue Parameters**		
	**Individual Culture**		**Co-Culture**		**Co-Culture Composition (%) ^#^**		Rescue Index *
**Condition**	***exoY***	***exoYexpA***	***exoY***	***exoYexpA***	***exoY***	***exoYexpA***	
Control	2.04 × 10^9^	6.04 × 10^2^	3.24 × 10^9^	1.25 × 10^9^	72.16	27.84	2.07 × 10^6^
As 100	1.73 × 10^8^	4.13 × 10^2^	3.30 × 10^8^	2.05 × 10^8^	61.68	38.32	4.96 × 10^5^
Hg 20	2.67 × 10^8^	2.35 × 10^2^	2.91 × 10^8^	2.34 × 10^8^	55.43	44.57	9.96 × 10^5^

Mean values of three different assays performed in triplicate per treatment correspond to cfu·mL^−1^ of planktonic or sessile cells after 48 h of growth on TY medium (control), TY medium with NaAsO_2_ 100 µM (As 100) and TY medium with HgCl_2_ 20 µM (Hg 20). Values of standard deviation are omitted to simplify the display and formatting of the table. A two-log difference in bacterial counts involves statistically significant differences from each other according to Fisher’s LSD test (*P* < 0.05). **^#^** cfu·mL^−1^ of *exoY* or *exoYexpA* × 100/TC. TC: Total count (cfu·mL^−1^ *exoY* + cfu·mL^−1^ *exoYexpA*). * cfu·mL^−1^ *exoYexpA* strain in co-culture/cfu·mL^−1^ *exoYexpA* in individual culture.

**Table 5:** Bacterial strains used in this study.

*S. meliloti* Strains	Relevant Properties	Reference
Rm8530	SU47 str21 expR101 (expR^+^)	[9]
Rm8530 *exoY*	Rm8530 exoY210::Tn5, Neo^R^	[16]
Rm8530 *expA*	Rm8530 expA3::Tn5–233, Gm^R^	[16]
Rm8530 *exoYexpA*	Rm8530 exoY210::Tn5, expA3::Tn5-233 Neo^R^ Gm^R^	[16]

The Rm8530 is the wild type (WT) strain, capable of synthesizing EPS I and EPS II. The Rm8530 *exoY* strain is a mutant incapable of producing EPS I. The Rm8530 *expA* strain is a mutant defective in biosynthesis of EPS II. The Rm8530 *exoYexpA* strain is a double mutant incapable of producing both, EPS I and EPS II.
